# Immunisation with BCG in the Maringue District, Sofala Province, Mozambique

**DOI:** 10.1155/2013/312065

**Published:** 2013-04-30

**Authors:** Dario Consonni, Marina Margarida Montenegro Agorostos Karagianis, Giuseppe Bufardeci

**Affiliations:** ^1^Epidemiology Unit, Department of Preventive Medicine, Fondazione IRCCS Ca' Granda—Ospedale Maggiore Policlinico, Via San Barnaba 8, 20122 Milan, Italy; ^2^Sofala Province Health Directorate, Beira, Mozambique; ^3^AISPO, Beira, Mozambique

## Abstract

*Objectives*. We evaluated immunisation with Bacille Calmette-Guérin (BCG) among newborns in 2011 in the Maringue District, Sofala Province, Mozambique, which includes seven health units. The study was motivated by the fact that in official reports, immunisation coverage was unreliable (more than 100%). *Methods*. The office of maternal-child health of the central Maringué-Sede health unit provided the number of live newborns in 2011 at the maternal clinics of the seven health units and an estimate of the number of home deliveries. From vaccination registers, we abstracted records of BCG vaccinations administered in the period 01/01/2011–30/06/2012 to children born in 2011. *Results*. The number of live newborns was 3,353. Overall, the number of BCG vaccinations administered was 2,893, with a coverage of 86.3%. *Conclusion*. In this study, we could only calculate an approximate coverage estimate, because of unavailability of adequate individual information. Recording practices should be changed in order to allow use of individual information and linkage across different information sources and thus a more precise vaccination coverage assessment.

## 1. Introduction

In Mozambique, a nationwide immunisation program (Extended Programme of Immunisation, EPI; in Portuguese: Programa Alargado de Vacinaçao, PAV) was started in 1979 [1]. Since performance was poor, in 2005, a comprehensive Multi Year Plan (cMYP) was launched with a view to devising strategies in line with the WHO/UNICEF Global Immunisation Vision and Strategy (GIVS) [2] with the objective to reach at least 90% national vaccination coverage and at least 80% coverage in every district. The current immunisation schedule includes Bacille Calmette-Guérin (BCG) at birth; oral polio vaccine (OPV) at birth, 6, 10, and 14 weeks; Diphtheria, Pertussis, Tetanus Hepatitis B (DTP-HepB) at 6, 10, and 14 weeks; measles, at 9 months [1].

**Figure 1:**
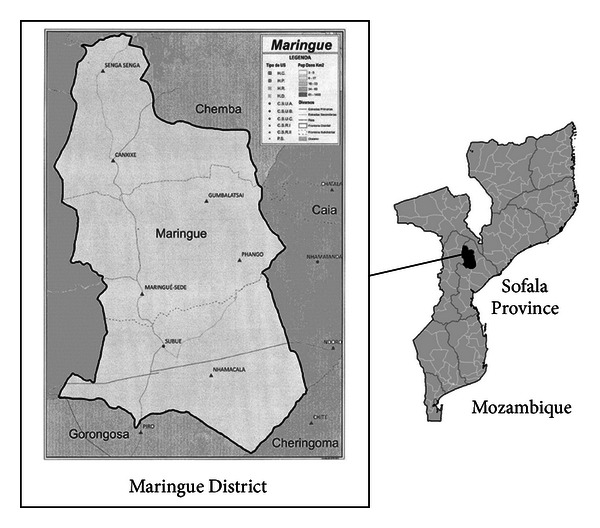
Map of the Maringue District indicating the location of the seven health units (Unidade Sanitaria, US): Maringué-Sede, Canxixe, Gumbalatsai, Nhamacala, Phango, Senga-Senga, and Subue.

In reports from the Ministry of Health (2004–2008), immunisation coverage percentages were largely variable across years and sometimes unreliable (>100%) [1]. The reasons for these anomalous figures are at least two. (1) The denominator (i.e., the number of children <1 year, so-called “grupo alvo,” target group) is only presumed. In fact, it is calculated by taking a fixed percentage (4%) of the whole population (almost 23 million people in 2011-2012) [2]. (2) The reference periods of immunisation and birth data are different; that is, part of the immunisation doses administered in a given year are given to infants born in the previous year. Also, the WHO website on Mozambique reports highly variable and unusually high (>99.5%) percentages [3]. The 2010 estimates were as follows: BCG: 90%; DPT-HepB, third dose: 74%; OPV, third dose: 73%; measles: 70%. Similarly, in the official reports of the Maringue District, Sofala Province, which includes seven health units (Unidade de Saùde, US) (Figure 1), BCG coverage in 2011 was clearly unreliable (108.6%). The reasons are the same seen previously for the national coverage. In the district, the target group is estimated as 4% × 84,719 (the population of the district) = 3,389.

Associazione Italiana per la Solidarietà tra i Popoli (AISPO), Milan, Italy, is a nongovernmental organisation recognised by the Italian Ministry of Foreign Affairs [4]. In 1995, AISPO started operating in Mozambique, Sofala Province. The activities include construction/extension of health centres, support for mobile teams to perform vaccinations on the field, and professional training. To get reliable immunisation coverage estimates, AISPO decided to make an independent assessment of BCG immunisation coverage in the Maringue District. BCG is an important vaccine, not only because of its protective efficacy against serious childhood tuberculosis, but also as “the gateway” to EPI and child targeted health packages. In this paper, we describe the project and report results regarding BCG coverage. The kind of study we are illustrating can be useful to periodically and independently test the method proposed by the WHO and used by the Ministry of Health. However, we also document difficulties in obtaining precise coverage estimates based on current recording practices.

## 2. Methods

After field surveys and recognition of the available information, we realized that a study using individual records was not feasible. Therefore, we opted for a study using routinely collected grouped data. Since this study was retrospective and did not involve individual information, ethical approval was not required.

### 2.1. Identification of Live Newborns

The central Maringué-Sede health unit provided (1) the number of live newborns in 2011 at the seven maternal clinics in each health unit, recorded in the birth registers (Registo de Maternidade); (2) an estimate of the number of home deliveries. We verified the reliability of the recorded number of alive newborns by consulting the birth registers of three health units (Maringué-Sede, Canxixe, and Phango). Agreement was good—our estimate: 1,863; estimate made by the central Maringué-Sede: 1,840. 

### 2.2. Identification of Vaccinated Children

Each health unit keeps two registers of vaccinations (Livros de Registo de Vacinaçao de Crianças): “Posto Fixo” for vaccinations performed at the health unit and “Brigada Mòvel” for immunisation campaigns performed in the territory with mobile teams. The registers contain information on sex and date of birth of the newborn and dates of vaccination for each type/dose of vaccine. Place of birth is not recorded; so, they do not allow to identify the infants born at the health unit and those born at mother's home. Note that when a vaccine dose has been administered in another health unit, a simple tally sign (*✓*) is written, instead of the date. From these registers, we counted the number of BCG vaccinations actually administered (i.e., with a date recorded in the register, omitting vaccinations marked with a tally to avoid multiple counting) to children born in 2011. Although BCG should be administered at birth, this is not always the case, for several reasons, including children born in the community; temporary or permanent unavailability of BCG vaccines because of logistic problems (refrigerator failures or lack of stored vaccines). Therefore, in order to catch late vaccinations, we recorded all vaccinations administered through June 2102.

### 2.3. Statistical Analysis

The proportion of infants vaccinated with BCG was calculated by dividing the number of BCG doses administered in 2011 and in the first half of 2012 by the number of newborns in 2011. The 95% confidence interval of the proportion was calculated using the Agresti-Coull formula [5] available in the software Stata 12 [6].

## 3. Results

The numbers of children born in 2011 and of BCG doses administered in 2011–2012 are shown in Table 1. We provide detailed results to give a picture of the births and of vaccination activities in the district. However, calculation of BCG coverage at the health unit level makes little sense, because children resident in the territory of a given health unit may be vaccinated elsewhere for several reasons. For example, in the Canxixe health unit, the refrigerator when vaccines were stored failed in 2011, with a dramatic decrease of the percentage of vaccinated children (130/488 = 26.6%). Logistic problems have probably occurred also in the Gumbalatsai, which also shows a very low coverage (49/221 = 22.2%). The newly established Phango health unit started vaccination activities in April 2011. The other health units somehow compensated for these problems. For example, in Maringué-Sede, the number of BCG vaccinations (1,436) was larger than the number of newborns (1,270).

The number of newborn deliveries in the whole district was 3,353, of which 3,059 (91.2%) at the maternal clinic of the health unit and 294 (8.8%) in the community. Percentages of home deliveries across health units ranged from 0.4% to 19.1%. In the study period, only the Maringué-Sede health unit performed BCG vaccinations campaigns with mobile teams on the territory (100 doses administered in 2011 and 63 in the first half of 2012). The total number of administered doses was 2,629 (90.9%) in 2011 and 264 (9.1%) in the first half of 2012. The number of BCG vaccinations performed in the seven health units was 2,893. BCG coverage was 2,893/3,353 = 86.3% (95% confidence interval: 85.1–87.4). 

## 4. Discussion

In the period 01/01/2011–30/06/2012, we estimated in the Maringue District a BCG coverage of 86.3% for children born in 2011. This figure is only approximate because we could not rely on carefully recorded individual data. From BCG results, we can plausibly infer that for other vaccinations the coverage percentages are lower, because they require several doses administered weeks to months after delivery. In fact, also national data shows a lower coverage for the other vaccinations [3]. 

In interpreting the result, we must take into account several implicit limits in the use of grouped data. First, the number of home deliveries was obtained based on children visits after birth at the health unit. This number might have been underestimated. In a worst-case scenario, assuming an underestimate of 50% (i.e., 294 more children born in the community) the BCG coverage would be 79.3%. Second, part of the babies could have been vaccinated later, in the second half of 2012. However, this number is likely to be quite small, considering that most children (90.9%) have been vaccinated in their birth year (2011). Third, some children (especially those living near the borders of the Maringue District) may have been vaccinated in nearby districts. On the other hand, a reversal flow of children living outside may have been vaccinated within the Maringue District. Unfortunately, we were unable to estimate the net balance because we could not rely on individual data.

Before carrying this study using grouped data, we had considered other design options. The first option was a classical cross-sectional sample survey called “30 × 7,” which involves the enrolment of at least 7 children in 30 randomly selected villages [7]. This type of study, suggested by WHO in developing countries, would also allow to perform a BCG scar counting (that would give a direct picture of the quality of immunisation). Unfortunately, this was not feasible for two main reasons: lack of demographics data and the very large number of small family units spreading over a territory of several tens of kilometres. The second option, a cohort study using recorded individual data, was also infeasible because data linkage was not possible. In fact, the register of births only records the name of the mother, while the vaccination register initially records mother's name and at following vaccination visits the child's name. Availability of individual information would have allowed identification of children born outside and inside the district. Moreover, individual information would be especially important to identify the unvaccinated children and the reasons for noncompliance and finally to target interventions.

The deficiencies in the process of immunisation data collection and reporting that we described earlier were also noted by other studies performed in Mozambique. A study evaluated routine surveillance data as a tool to investigate measles outbreaks in Maputo (1998) and Manica Province (2002) [8]. It was concluded that the reporting system did not provide the data needed by EPI managers to make evidence-based decisions and precluded in-depth analysis to monitor measles epidemiology in the country. An improvement of routine surveillance and health information systems (HISs) was therefore recommended. A study carried out in 2003 in the Nassa Province, Cuamba District, evaluated practices of record keeping and reporting of immunisation data by means of semistructured interviews, participant observation, and review of the data collection materials [9]. The authors concluded that in “Mozambique and other country settings […] poor data quality constitutes a bottle neck for good information systems as well as good decision making.” More recent evaluations (November 2007 through October 2008) in three districts (Beira City, Dondo, and Caia) of the Sofala Province found that HISs were sufficiently reliable and consistent, supporting their use in primary health care program monitoring and evaluation [10]. On the other hand, the authors underlined the need to enhance the design, testing, and subsequent use of operational research tools to further expand the quality and use of routine data by health managers and policymakers. 

## 5. Conclusion

In this study, using routinely collected information, we estimated a BCG coverage of 86.3% in the Maringue District. This figure is only approximate because of the unavailability of adequate individual information. Our study also underlines deficiencies of current recording practices, which should be changed in order to allow use of individual information and linkage across different information sources (preferably using computerised archives) and thus a more precise assessment of vaccination coverage. We concur with the authors of a recent editorial that “a major challenge for […] Mozambique is to strengthen local capacity for data collection, management, and analyses” [11]. 

## Figures and Tables

**Table 1:** Number of children born in 2011 and of Bacille Calmette-Guérin (BCG) vaccinations administered to them in the period 01/01/2011–30/06/2012 in the Maringue District, Sofala Province, Mozambique.

Health unit (period of BCG data collection)	Children born alive in 2011			BCG vaccinations		
	At the health unit	Home deliveries	Total	Year 2011	Year 2012	Total
	*N*%	*N*%	*N*%	*N*%	*N*%	*N*%
Maringué-Sede	1,172	98	1,270	1,303	133	1,436
(01/01/2011–14/05/2012)	92.3	7.7	100	90.7	9.3	100
Canxixe	452	36	488	123	7	130
(01/01/2011–14/05/2012)	92.6	7.4	100	94.6	5.4	100
Gumbalatsai	185	36	221	44	5	49
(01/01/2011–30/06/2012)	83.7	16.3	100	89.8	10.2	100
Nhamacala	293	7	300	267	16	283
(01/01/2011–30/06/2012)	97.7	2.3	100	94.3	5.7	100
Phango	216	45	261	208	21	229
(19/04/2011–14/05/2012)	82.8	17.2	100	90.8	9.2	100
Senga Senga	444	2	446	342	64	406
(01/01/2011–30/06/2012)	99.6	0.4	100	84.2	15.8	100
Subue	297	70	367	342	18	360
(01/01/2011–30/06/2012)	80.9	19.1	100	95.0	5.0	100

Total	3,059	294	3,353	2,629	264	2,893
	91.2	8.8	100	90.9	9.1	100
